# Long-term cortisol secretion in attention deficit hyperactivity disorder: roles of sex, comorbidity, and symptom presentation

**DOI:** 10.1007/s00787-023-02180-1

**Published:** 2023-03-14

**Authors:** Ursula Pauli-Pott, Nadine Skoluda, Urs M. Nater, Katja Becker, Friederike Derz, Elena Kaspar, Daria Kasperzack, Kira Kehm, Marie Kött, Christopher Mann, Pia Schurek, Wilfried Pott, Susan Schloß

**Affiliations:** 1 Department of Child and Adolescent Psychiatry, Psychosomatics and Psychotherapy, Philipps-University of Marburg, Schützenstrasse 45, 35039 Marburg, Germany; 2 Clinical Psychology, Department of Clinical and Health Psychology, Faculty of Psychology, University of Vienna, Liebiggasse 5, 1010 Vienna, Austria; 3 Research Platform The Stress of Life (SOLE)—Processes and Mechanisms underlying Everyday Life Stress, Vienna, Austria; 4 Center for Mind, Brain and Behavior (CMBB), University of Marburg Justus Liebig University Giessen, Hans-Meerwein-Straße 6, 35032 Marburg, Germany

**Keywords:** Hypothalamic–pituitary–adrenal axis dysregulation, Hair cortisol concentration, Sympathetic reactivity, Attention deficit hyperactivity disorder, Oppositional defiant disorder

## Abstract

Low activity of the hypothalamic–pituitary–adrenal axis (HPAA) has been found in children with attention deficit hyperactivity disorder (ADHD). The condition may be related to the reduced attention regulation capacity and/or to comorbid oppositional defiant or conduct disorder (ODD/CD). Sex differences are probable but not sufficiently studied. We analyzed the HPAA activity and sympathetic nervous system reactivity (SR) in children with ADHD while accounting for ADHD symptom presentation, comorbidity, and sex differences. The sample comprised 205 children, 98 (61 boys, 37 girls) with ADHD and 107 (48 boys, 59 girls) healthy controls. DSM-5 phenotypic symptom presentation and comorbid ODD/CD were assessed using clinical interviews. Hair cortisol concentration (HCC) was used to assess the long-term, cumulative activity of the HPAA. SR was assessed via skin conductance response (SCR). For control purposes, comorbid internalizing symptoms and indicators of adverse childhood experiences (ACE) were assessed. Children were medication naive. Boys presenting with predominantly inattentive symptoms (ADHD-I) showed lower HCC than healthy boys. Girls presenting with combined symptoms (ADHD-C) showed higher HCC than did healthy girls (*p*’s < 0.05, sex-by-group interaction, *F* (2,194) = 4.09, *p* = 0.018). Boys with ADHD plus ODD/CD showed a blunted SR (*p* < 0.001, sex-by-group interaction, *F* (2,172) = 3.08, *p* = 0.048). Adjustment for ACE indicators led to non-significant differences in HCC but did not affect differences in SR. HCC constitutes an easily assessable, reliable, and valid marker of phenotypic ADHD-related features (i.e. symptom presentation and comorbidity). It indicates more homogenous subgroups of ADHD and might point to specifically involved pathophysiological processes.

## Introduction

Hypothalamic–pituitary–adrenal-axis (HPAA) function has been extensively studied in attention deficit hyperactivity disorder (ADHD) and the broader domain of externalizing disorders (comprising e.g. oppositional defiant disorder (ODD) and conduct disorder (CD)), with meta-analyses of this research revealing a lower basal cortisol level (in serum and saliva samples) in children and adolescents with ADHD. However, considerable heterogeneity in the effects of individual studies has been found [1]. A meta-analysis by Kamradt et al. [2] summarized studies on cortisol reactivity to stress exposure. The authors reported that children/adolescents with ADHD did not differ from healthy controls, but again, heterogeneity was substantial. Reviews focusing on ODD/CD concluded that the blunted cortisol response sometimes observed in children/adolescents with ADHD is most likely due to the often comorbid ODD/CD [3, 4]. Research on HPAA function in ADHD has too seldom considered influences of comorbidity and other potential moderator variables such as past and present medication use, sex differences, phenotypic symptom presentation, and environmental conditions which might explain the observed heterogeneity.

ADHD is a highly heritable but heterogeneous disorder, with high variation in the phenotypic symptom presentation. Accordingly, the DSM-5 distinguishes between the presentation of predominantly inattentive symptoms, predominantly hyperactive/impulsive symptoms, and a combined presentation [5]. In about two-thirds of affected children, ADHD is accompanied by externalizing disorders (e.g. ODD and CD) and/or internalizing disorders (anxiety, depressive disorders) [6]. Moreover, considerable sex differences exist with regard to prevalence rates, symptom presentation, and comorbid disorders [7]. Given that HPAA function also differs between sexes [8], shows heritability [9] and adjusts to environmental conditions [10], and is related to neurocognitive and emotional functions (e.g. [11, 12]), it would be rather surprising to find a uniform pattern of HPAA dysregulation in children with ADHD.

Low HPAA activity and reactivity in externalizing disorders (i.e. ADHD, ODD, and CD) have been explained by different theoretical concepts focusing on low autonomic and cortical arousal (linked via the locus coeruleus and the brain norepinephrine system). Concepts emphasizing low anxiety and low responsivity to rewarding stimuli in externalizing disorders refer to low arousal as an aversive physiological state that the individual attempts to normalize (increase) by seeking sensation through impulsive, risky, and rule-breaking behaviors [13]. Low anxiety has been suggested to lead to inadequate social learning because children do not fear the negative consequences of their behaviors [14, 15]. Accordingly, low sympathetic reactivity (e.g. measured through the skin conductance) has consistently been found to be associated with ODD/CD [16]. Other concepts emphasize cognitive dysfunction and propose that low arousal in ADHD primarily reflects a reduced capacity to adjust activation to environmental demands [17, 18]. Inattention and poor executive functioning have been thought to result from too low and less-regulated energetic state [19]. As the autonomic nervous system and the HPA system adjust to environmental demands by coordinated, mutually supporting action [10], both concepts (i.e. ODD/CD-related low autonomic arousal/reactivity to emotional stimuli and attention deficit-related low capacity to adjust activation to cognitive demands) might explain a reduced HPAA activity. However, it is an open question whether ADHD-related low cortisol secretion actually associates with comorbid ODD/CD and/or attention deficits.

Previous research by our group provided support for the cognitive activation concept. We used the hair cortisol concentration (HCC) to assess the long-term, cumulative activity of the HPAA in medication-naïve 4–5-year-old children. The HCC has proven to validly capture the accumulated, integrated long-term activity of the HPAA [20–22]. Compared to blood and salivary cortisol measures, which reflect the cortisol secretion only at a single time point, HCC shows increased stability/reliability of measurement. The non-invasive procedure makes hair samples both independent form acute stress influences and well-acceptable for participating children [23].

We found that low HCC was associated with ADHD-inattentive symptoms in particular, as well as with poor working memory performance [24, 25]. Moreover, HCC at the age of 4–5 years predicted ADHD development by the age of 8 years [26]. These links were more pronounced in boys than in girls or referred exclusively to boys, and the results remained unchanged after controlling for comorbid symptoms of ODD/CD and anxiety/depressive disorders [24–26]. Very few studies have analyzed HPAA function in ADHD while accounting both for the phenotypic symptom presentation and for comorbid symptoms of ODD/CD and anxiety/depressive disorders. Isik et al. [27] studied drug-naïve children presenting with combined (inattentive and hyperactive) symptoms. Serum cortisol did not differ between these children and healthy controls. Angeli et al. [28] reported a lower cortisol awakening response and diurnal profile (using saliva samples) in children with ADHD than in healthy controls, irrespective of the presentation of predominantly inattentive symptoms or combined symptoms. The latter study excluded children with comorbid disorders. On the whole, it is currently unclear whether the low cortisol level of children with ADHD indicates low attention regulation capacity and/or low emotional reactivity associated with comorbid ODD/CD. In the present study, we, therefore, consider comorbid ODD/CD and assess the sympathetic reactivity to mildly anxiety-eliciting stimuli.

ADHD is more common in families with a low socioeconomic status [29] and is closely related to psychosocial risk factors including maternal depression/depressive symptoms [30, 31]. As these conditions have sometimes been found to be associated with HCC in the general population [20, 32] it is possible that low HCC in ADHD indicates exposure to environmental adversity. Therefore, we control for influences of indicators of adverse childhood experiences (ACE). Moreover, we aimed to investigate a sample of medication-naïve children with ADHD, as medication (e.g. with stimulants) may affect HPAA function [33]. Given that low HPAA activity can be assumed to be related to a reduced capacity to regulate arousal and attention, we sought to compare children presenting with predominantly inattentive symptoms (ADHD-I type), children with combined inattentive and hyperactive/impulsive symptoms (ADHD-C type), and healthy control children with regard to HCC. We hypothesized a moderator effect by sex of child (i.e. a sex-by-group interaction effect) on HCC, with boys of the ADHD-I type showing the lowest HCC. As low sympathetic reactivity to anxiety-eliciting stimuli of children with comorbid ODD/CD might also explain the low HPAA activity found in children with ADHD, it is further hypothesized that children with ADHD and comorbid ODD/CD show lower HCC and lower sympathetic reactivity than healthy control children.

## Methods

### Participants

The complete study sample consisted of 290 children aged between 6 and 11 years. Of these children, 130 (92 boys) were diagnosed with ADHD. Children were medication-naïve (i.e. never received any continuous psychotropic medications). Further exclusion criteria were: IQ < 80, motor disabilities, sensory disabilities, chronic physical diseases involving brain functions, any continuous pharmacological treatment in the last three months (including topical and inhaled steroids), and insufficient German language skills of parents or child. Children were recruited via a child and adolescent psychiatry practice and outpatient clinics in Gießen, Marburg, and Butzbach (Middle Hesse, Germany). Healthy control children were from the same district. The control children took part in the school-age assessment wave of a longitudinal study (see e.g. Pauli-Pott et al. [26]) or were recruited through primary schools in Marburg. In addition to the exclusion criteria used for the ADHD group, children with a diagnosis of any mental disorder were excluded.

HCC was analyzed in 205 children (107 healthy control children and 98 children diagnosed with ADHD). The remaining children (*n* = 75 excluded because parents refused to take part in the hair collection part of the study or the child did not fulfill the criterion of minimum hair length of 3 cm; *n* = 6 because insufficient hair was collected; *n* = 4 were outliers in the HCC distributions, see below) did not differ from those with complete data regarding ADHD vs. control group membership (*χ*^2^ (1) = 2.38), maternal and paternal education level (*χ*^2^ (4) scores were 2.24 and 2.54, respectively), and ODD/CD, anxiety and depressive disorder symptom scores (*t*-scores between 1.59 and − 1.07). Significantly more boys than girls were excluded from the analysis due to the shorter haircuts of the boys (*χ*^2^ (1) = 19.52, *p* < 0.001). However, in all further analyses, sex is taken into account.

Table 1 contains descriptive data of the samples with complete data. Parents and children gave their written informed consent to participate in the study and received an expense allowance of 30 Euros. The study was approved by the Ethics Committee of the Medical Faculty, University of Marburg.

**Table 1:** Description of samples

	ADHD group	Control group	Statistical tests*
Sex	*n* (%)	*n* (%)	
Male	61 (62.9)	48 (44.9)	*χ*^2^ (1) = 6.25 *p* = 0.012
Female	37 (37.1)	59 (55.1)	
Age in years	m (s, range)	m (s, range)	
	8.58 (0.90; 6–11)	8.88 (1.34; 6–11)	*t* = 1.94 *p* = 0.054
Psychosocial adversity index	*n* (%)	*n* (%)	
High	62 (63.3)	41 (38.3)	*χ*^2^ (1) = 11.76 *p* = 0.001
Low	36 (36.7)	66 (61.7)	
Education level of mother	*n* (%)	*n* (%)	
No compl./basic education	19 (19.8)	5 (4.7)	*χ*^2^ (3) = 12.73 *p* = 0.005
Vocational qualification	30 (31.3)	40 (37.7)	
High school	23 (24.0)	22 (20.8)	
University	24 (25.0)	39 (36.8)	
(No reply)	2	1	
Education level of father	*n* (%)	*n* (%)	*χ*^2^ (3) = 8.03 *p* = 0.045
No compl./basic education	24 (25.0)	18 (17.5)	
Vocational qualification	34 (35.4)	24 (23.3)	
High school	16 (16.7)	22 (21.4)	
University	22 (22.9)	39 (27.9)	
(No reply)	2	4	
	m (s, range)		
ODD/CD symptom count (CAPA, clinical parent interview)	2.97 (2.11, 0–8)	1.19 (1.20, 0–3)	*t* = 7.39 *p* < 0.001
Anxiety symptom score (DISYPS, clinical parent interview)	3.12 (3.38, 0–16)	1.58 (1.86, 0–8)	*t* = 3.65 *p* < 0.001
Depressive symptom score (DISYPS, clinical parent interview)	2.90 (3.04, 0–14)	1.10 (1.49, 0–5)	*t* = 4.98 *p* < 0.001

*ODD/CD* oppositional defiant disorder/conduct disorder, *compl* completed, *m* mean, *s* standard deviation, *ADHD* attention deficit hyperactivity disorder

*Test statistics are reported for descriptive purposes

### Variables

#### ADHD symptom presentation groups

The ADHD diagnostic module of the Child and Adolescent Psychiatric Interview (CAPA) by Angold et al. [34] in the German-language DSM-5 version (translated by Dr. Yvonne Otto, Child and Adolescent Psychiatric Clinic, University of Leipzig) was conducted with the mothers of all children to confirm the presence or absence of an ADHD diagnosis. The CAPA is a well-validated, widely established clinical interview that allows clinical diagnoses to be made according to the DSM-5.

Of the 98 children with ADHD, 59 children (41 boys) presented with combined inattentive and hyperactive/impulsive symptoms according to the DSM-5 (ADHD-C) and 35 children (17 boys) presented with predominantly inattentive symptoms (ADHD-I). The remaining four children (three boys) presented with predominantly hyperactive/impulsive symptoms and were excluded.

#### Hair cortisol concentration (HCC)

Several thin hair strands were cut from the posterior vertex region of the head. The first proximal scalp-near 3-cm segment was used for the determination of HCC. This 3-cm segment is thought to reflect the cumulative cortisol secretion of the past 3 months. Hair-washing and cortisol extraction procedures were based on a laboratory protocol first described by Stalder et al. [35], with minor modifications [36, 37]. The intra-assay and inter-assay coefficient of variance (CV) of the immunoassay were below 5% (1.9% and 4.6%, respectively). In the whole sample, the HCC showed a skewed distribution. The distributions were therefore normalized by the exclusion of outliers exceeding the mean + 3 SD (four cases) and subsequently log-transformed.

Potential influences of the child’s age, hair wash frequency, BMI z-score, and family socioeconomic status (SES) (indicated by maternal education level) on the HCC scores (see [11, 38]) were checked in the subsamples of ADHD and healthy children. We found no significant correlations of HCC with the age of the child (ADHD group: 0.00; control group: 0.03), the hair-washing frequency (− 0.02; − 0.03), and the BMI z-score (0.07, 0.10). Maternal education level related to HCC in the ADHD group (0.21, *p* < 0.05) but not in the control group (0.01). We controlled for SES variables in the main analyses.

#### Sympathetic reactivity

Reactivity of the sympathetic nervous system can be validly measured by indices of electrodermal activity (EDA) [39]. We measured the electrodermal reactivity to six questions from the interview-on-attractive-toys task (measuring withdrawal vs. approach behavior; adapted from Asendorpf, [40]). The child is told that he/she will receive a gift for participating, but that before receiving the gift, he/she will take part in a video-recorded interview, conducted by a colleague, on the attractiveness of a series of toys. After 3 min of waiting, an unfamiliar adult enters the room, places six different toys in front of the child, and asks six questions, with a break of 10 s between the child’s answer and the next question. The procedure was videotaped, and video and EDA recordings were synchronized. The measurement of EDA followed the guidelines by Boucsein et al. [39] using a BioPac MP150 system with two silver-silver chloride (Ag/AgCl) disposable electrodes attached to the middle phalanges of the middle and ring finger of the non-dominant hand. The frequency of the skin conductance responses (SCR; in microsiemens) elicited by the six questions was used as an indicator of the child’s sympathetic reactivity.

#### Comorbidity

##### ODD and CD

The ODD and CD diagnostic modules of the CAPA interview were conducted with the mother. Of the 98 children with ADHD, *n* = 36 (25 boys) children received a diagnosis of ODD and *n* = 5 (three boys) received an additional diagnosis of CD. Dimensional ODD/CD scores were used to capture the whole range of symptoms in children from the ADHD and the control group.

Furthermore, we used three scales from the German-language Parent Rating Scale for Oppositional Defiant and Conduct Disorder (FBB-SSV) [41]): the oppositional symptoms scale, the conduct disorder symptoms scale, and the callous-unemotional scale. This questionnaire is suitable for the assessment of ODD/CD symptoms and callous-unemotional (CU) traits in line with the DSM-5 and ICD-10 and has shown good psychometric properties.

##### Anxiety and depressive disorder symptoms

The clinical screening interview of the Diagnostic System of Mental Disorders in Children and Adolescents (DISYPS) [41] was conducted with the mother by a trained psychologist. The interview assesses central symptom criteria (according to the DSM-5) for anxiety and depressive disorders. The anxiety and depressive disorder symptom scores were used to capture symptom expression in each domain.

#### Adverse childhood experiences

For control purposes, maternal education level, psychosocial risks, and maternal depressive symptoms were assessed. Maternal education level and psychosocial risks (according to Laucht et al. [42], index comprising the presence of: at least one parent without occupational qualification, at least one parent with a broken home background, early parenthood, parental separation, and unwanted pregnancy) were assessed by structured interviews. Maternal depressive symptoms were measured using the German version of the Center for Epidemiologic Studies Depression Scale (CES-D). For this version, good internal consistency (Cronbach’s Alpha = 0.89) and validity (correlations with other depression questionnaires) have been established [43].

### Statistical analysis

To test our first hypothesis, analysis of variance (ANOVA, model 1) was conducted with HCC as criterion and sex and group as between-subjects factors. The four ADHD-H-type children were excluded from this analysis. In subsequent analyses, we adjusted for the CAPA ODD/CD symptom interview score and the oppositional symptoms scale, the conduct disorder symptoms scale, and the callous-unemotional scale of the FBB-SSV Parent Rating Scale in model 2, for anxiety and depressive disorder symptom scores of the DISYPS interview in model 3, and for the indicators of ACE (i.e. maternal education, psychosocial risk score, and maternal depressive symptoms) in model 4. To test our second and third hypotheses regarding low HCC and SR of children with ADHD and comorbid ODD/CD analogous ANOVA models were used. We adjusted for anxiety and depressive disorder symptom scores (model 2) and for the indicators of ACE (model 3). Distributions of all psychopathological symptom dimensions were right-skewed in the control and the ADHD groups. As high scores probably represent an extreme expression of symptoms rather than artifacts, outliers (> m + 3 s) were winsorized and the distributions were then log-transformed, leading to sufficiently symmetric distributions with skewness coefficients below 1.0. The hypotheses were tested with an alpha error of 5% (level of significance of 0.05).

## Results

### ADHD symptom presentation

**Fig. 1:**
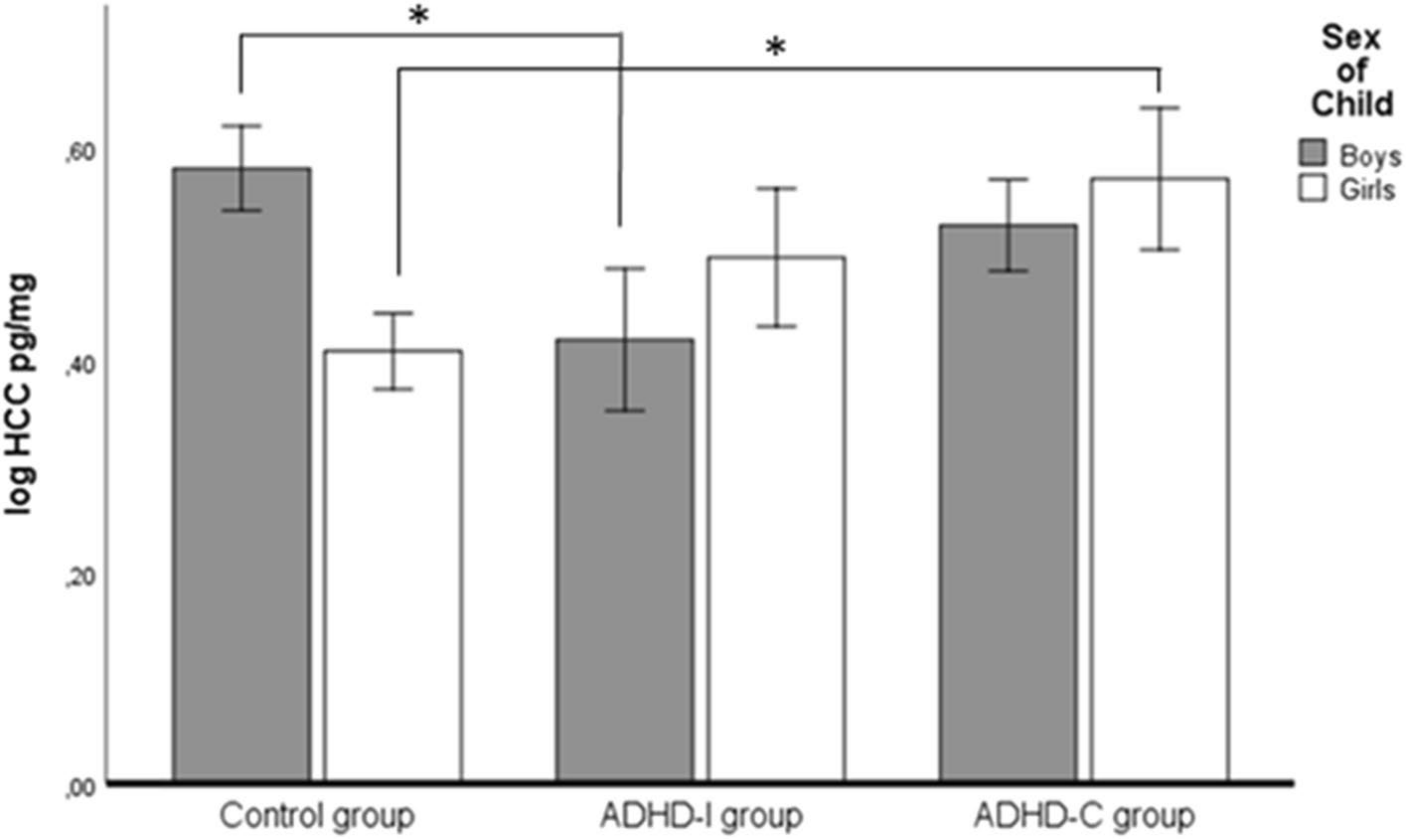
Significant interaction effect between sex and group (ADHD-I, ADHD-C vs. control group). Post hoc comparisons (Scheffé tests): in boys: control group > ADHD-I group; in girls: control group < ADHD-C group (*p < 0.05). Means (height of column) ± 1s_e_ are depicted; *ADHD*, attention deficit hyperactivity disorder, *ODD* oppositional defiant disorder, *CD* conduct disorder, *ADHD-I* ADHD with predominantly inattentive symptoms, *ADHD-C* ADHD with combined inattentive and hyperactive/impulsive symptoms, *HCC* hair cortisol concentration

The analysis of variance (ANOVA) with sex and group (i.e. ADHD-I, ADHD-C, and control group) as between-subjects factors revealed a statistically significant sex-by-group interaction effect on HCC (Table 2, Fig. 1). Consistent with our expectation, boys in the ADHD-I group showed the lowest HCC, with post-hoc comparisons revealing significantly lower HCC in ADHD-I than in control boys. Moreover, girls with ADHD-C showed significantly higher HCC than did girls in the control group (Fig. 1). Results remained after adjusting for the ODD/CD dimensional symptoms scores (Table 2, model 2) and symptoms of anxiety and depressive disorders (Table 2, model 3). Likewise, the exclusion of children with ODD/CD diagnoses from the analysis did not change the significant interaction effect (*F* (2,160) = 3.15, *p* = 0.046). Adjusting for the indicators of ACE led to a non-significant interaction effect on HCC (Table 2, model 4). In all models, the main effects were not statistically significant (Table 2). Groups did not differ in the SR measure (main effect sex, *F* (1,182) = 1.18, main effect group, *F* (2,182) = 2.15, sex-by-group interaction, *F* (2,182) = 2.29).

**Table 2:** Comparison of boys and girls with ADHD-I, ADHD-C, and control children regarding HCC

		ADHD-I group		ADHD-C group		Control group	
		Boys (*n* = 17)	Girls (*n* = 18)	Boys (*n* = 41)	Girls (*n* = 17)	Boys (*n* = 48)	Girls (*n* = 59)
**HCC (lg)**	x̄ (s)	**0.42 (0.28)**	**0.50 (0.21)**	**0.53 (0.29)**	**0.57 (0.28)**	**0.58 (0.23)**	**0.41 (0.32)**
HCC pg/mg*		*3.27 (2.53)*	*3.53 (1.86)*	*4.24 (3.42)*	*4.62 (3.42)*	*4.39 (2.65)*	*3.29 (2.63)*

	Sex	Group	Sex × group
**Model 1**	*F* (1,194) = 0.15 *ns *	*F* (2,194) = 1.18 * ns*	*F* (2,194) = 4.09 *p* = 0.018
Model 2	*F* (1,189) = 0.21 * ns*	*F* (2,189) = 1.20 * ns*	*F* (2,189) = 4.11 *p* = 0.018
Model 3	*F* (1,170) = 0.02 * ns*	*F* (2,170) = 1.53 * ns*	*F* (2,170) = 4.55 *p* = 0.012
Model 4	*F* (1,166) = 0.86 * ns*	*F* (2,166) = 0.82 * ns*	*F* (2,166) = 2.82 *p* = 0.063

In bold, results of the tests of the hypotheses

Model 1, analysis of variance (ANOVA); model 2, analysis of co-variance (ANCOVA), adjusted for ODD/CD symptoms (i.e. CAPA interview score, oppositional defiant symptoms score, callous-unemotional score, and conduct disorder score of the FBB-SSV questionnaire); model 3, ANCOVA, adjusted for anxiety and depressive disorder symptoms; model 4, ANCOVA, adjusted for maternal education level, maternal depressive symptoms, psychosocial risks

*HCC* hair cortisol concentration,* ns* not statistically significant

*Means and standard deviations (in italics) of the original (not transformed) distribution are reported for descriptive purposes

### Comorbid ODD/CD

**Fig. 2:**
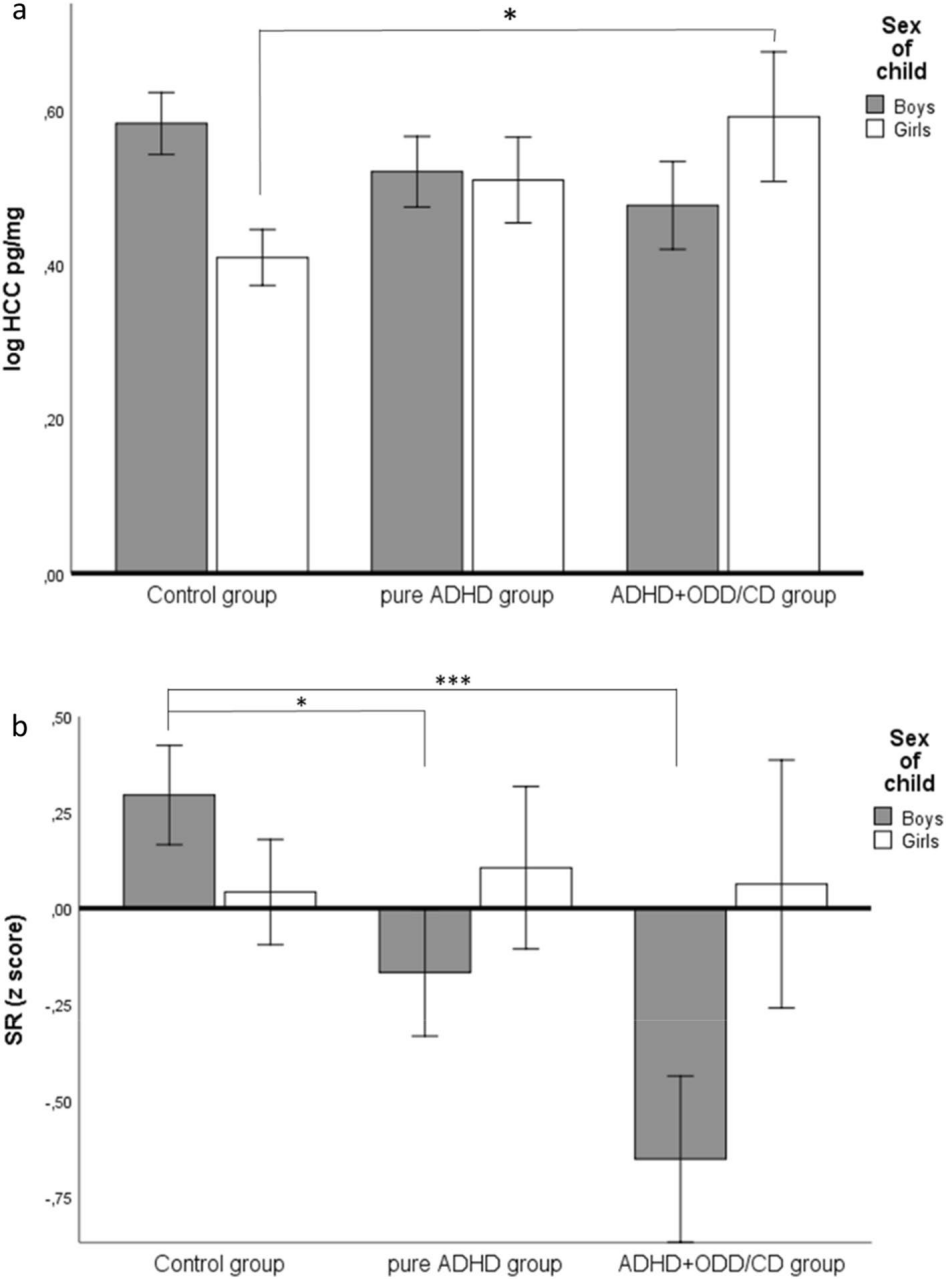
Significant interaction effect between sex and group (pure ADHD, ADHD + ODD/CD vs. control group) on HCC (**a**) and SR (**b**). Post hoc comparisons (Scheffé tests): **a** for girls: control group < ADHD + ODD/CD group, **b** for boys: control group > pure ADHD group and control group > ADHD + ODD/CD; *p < 0.05, ***p < 0.001. Means (height of column) ± 1s_e_ are depicted, *ADHD* attention deficit hyperactivity disorder, *CD* conduct disorder, *ODD* oppositional defiant disorder, *HCC* hair cortisol concentration, *SR* sympathetic reactivity

We compared boys and girls with pure ADHD, ADHD plus ODD/CD diagnosis, and healthy control children. The sex-by-group interaction effect on HCC proved to be statistically significant (Table 3, Fig. 2a). Post hoc tests revealed a significantly higher HCC in girls with ADHD + ODD/CD compared to healthy girls. Controlling for anxiety and depressive disorder scores did not change the results of this analysis (see Table 3, model 2). After adjusting for the indicators of ACE, the sex-by-group interaction effect was no longer significant (Table 3, model 3). In most models, the main effects of sex and group were not statistically significant (Table 3).

**Table 3:** Comparison of boys and girls with ADHD, ADHD + ODD/CD, and control children regarding HCC

		pure ADHD group		ADHD + ODD/CD group		Control group	
		Boys (*n* = 37)	Girls (*n* = 25)	Boys (*n* = 24)	Girls (*n* = 11)	Boys (*n* = 48)	Girls (*n* = 59)
**HCC (lg)**	x̄ (s)	**0.52 (0.28)**	**0.51 (0.24)**	**0.48 (0.30)**	**0.59 (0.25)**	**0.58 (0.23)**	**00.41 (0.32)**
HCC pg/mg*		*4.11 (3.23)*	3.80 (2.57)	3.82 (2.98)	4.58 (3.06)	4.39 (2.65)	3.29 (2.63)
**SR (z score)**	x̄ (s)	**− 0.17 (0.95)**	**0.11 (1.29)**	**− 0.65 (1.01)**	**0.06 (0.87)**	**0.30 (0.88)**	**0.04 (0.93)**
SR (% response)*		*56.19 (20.25)*	*62.02 (27.56)*	*45.83 (21.54)*	*61.11 (18.63)*	*66.07 (18.79)*	*60.67 (19.85)*

Effects		Sex	Group	Sex × Group
**Model 1**	**HCC (lg)**	*F* (1,198) = 0.26 ns	*F* (2,198) = 0.25 ns	*F* (2,198) = 3.81 *p* = 0.024
	**SR (z)**	*F* (1,185) = 2.09 ns	*F* (1,185) = 2.54 ns	*F* (1,185) = 3.08 *p* = 0.048
Model 2	HCC (lg)	*F* (1,172) = 0.10 ns	*F* (2,172) = 0.12 ns	*F* (2,172) = 3.96 *p* = 0.021
	SR (z)	*F* (1,167) = 5.26 *p* = 0.023	*F* (2,167) = 1.23 ns	*F* (2,167) = 6.52 *p* = 0.002
Model 3	HCC (lg)	*F* (1,171) = 1.86 ns	*F* (2,171) = 0.48 ns	*F* (2,171) = 1.86 ns
	SR (z)	*F* (1,172) = 2.52 ns	*F* (2,172) = 1.69 ns	*F* (2,172) = 3.66 *p* = 0.028

In bold, results of the tests of the hypotheses

Model 1, analysis of variance (ANOVA); model 2, analysis of co-variance (ANCOVA), adjusted for anxiety and depressive disorder symptoms; model 3, ANCOVA, adjusted for maternal education level, maternal depressive symptoms, psychosocial risks

*HCC* hair cortisol concentration,* SR* sympathetic reactivity, *ns* not statistically significant

*Means and standard deviations (s) (in italics) of the original, non-transformed distributions are reported for descriptive purposes

Regarding the SR measure, the sex-by-group interaction effect was statistically significant (Table 3, Fig. 2b). While no significant group differences emerged for the girls, boys with comorbid ADHD + ODD/CD and boys with pure ADHD showed lower SR than did healthy boys (Fig. 2b). Main effects were not statistically significant (Table 3). Results remained after adjusting for symptoms of anxiety and depressive disorders (Table 3, model 2) and indicators of ACE (Table 3, model 3).

The indicators of ACE explained variance in the HCC measure (i.e. adjustment for maternal education level, maternal depressive symptoms, and psychosocial risks led to non-significant results). To further explore this effect, we calculated the correlation coefficients of the ACE variables with HCC. In the ADHD group, maternal education level (*r* = 0.21, *p* = 0.046) and maternal depressive symptoms (*r* = − 0.22, *p* = 0.041) were significantly associated with HCC. Children of mothers with a lower education level and higher depressive symptoms showed lower HCC. The psychosocial risks did not correlate with HCC and in the control group of healthy children correlations between the ACE variables and HCC were not significant.

## Discussion

The present study aimed to identify phenotypic features associated with low HPAA activity in ADHD. Currently, it is not yet clear which components and associates of ADHD relate to the blunted HPAA activity of children with ADHD. In line with our hypotheses, significant sex differences emerged in the HCC-ADHD association (i.e. a significant sex-by-ADHD group interaction effect). As expected, boys presenting with predominantly ADHD inattentive symptoms (ADHD-I type) showed lower HCC than healthy boys. Girls with ADHD-C showed significantly higher HCC compared to healthy girls. The results remained unchanged after adjusting for comorbid symptom dimensions and after the exclusion of children with comorbid ODD/CD. Analyses of comorbid ODD/CD diagnoses revealed increased HCC in girls with ADHD plus ODD/CD compared to healthy girls. Boys with ADHD, and those with comorbid ADHD plus ODD/CD, showed reduced SR to the anxiety/withdrawal-eliciting stimuli. Adjustment for ACE indicators (i.e. maternal education level, maternal depressive symptoms, family psychosocial risks) led to non-significant HCC differences but did not affect the differences in SR. Taken together, the findings demonstrate that low long-term cortisol secretion is primarily a characteristic of boys with the ADHD-I type, while girls show a different pattern, characterized by relatively high cortisol secretion in those with the ADHD-C and the comorbid ADHD + ODD/CD type. Interestingly, ACE indicators explain these differences.

In general population samples, sex differences in HCC are well established, with females showing lower HCC than males [11]. In our study, the healthy girls showed lower HCC than the healthy boys on a descriptive level. This is well in line with the general population finding and indicates a good validity of our data. The low HCC in boys presenting with predominantly inattentive symptoms in the present study corresponds to previous findings of an association between low cortisol activity/reactivity and neurocognitive deficits [25, 44], the prediction of ADHD development by reduced HCC [26], and with theories suggesting a low capacity to adjust arousal to cognitive demands in children with ADHD [17, 19]. Moreover, attentional, cognitive control, and working memory deficits of children with ADHD involve structural and functional deviations in hippocampal and frontal brain circuitry [45]. Activity in these areas is known to be modulated by glucocorticoid secretion [12]. In cases of hyper- and hypocortisolism, impairing cortisol-mediated effects on cognition have been emphasized [46, 47]. Hence, investigation of the mechanisms linking HPAA dysfunction with ADHD subgroups could be worthwhile.

Low HCC in boys with ADHD was not related to comorbid ODD/CD. However, consistent with well-established models on ODD/CD [14–16], boys with comorbid ADHD + ODD/CD showed reduced SR to the anxiety/withdrawal eliciting stimuli. However, in comparison to the healthy boys the boys with pure ADHD also showed reduced SR. The latter result resembles previous findings of our group: in a community-based sample, ADHD symptoms were negatively associated with SR [48]. Other studies found hypoactivation of the sympathetic nervous system not only during cognitive but also during emotional tasks (though less consistent) in children with ADHD [17, 18]. This might explain our result. However, more research is needed to clarify the role of subclinical symptoms of ODD/CD regarding SR to emotional stimuli.

In the present study, girls with ADHD-C and with ADHD plus ODD/CD showed increased HCC compared to children in the control group. It might be speculated that hyperactive/impulsive and ODD/CD symptoms in girls reflect increased irritability and/or emerging depressive disorder symptoms. Irritability/depression is more prevalent in females [49, 50] and has been found to be associated with relative hypercortisolism [51]. However, this effect was not explained by the depressive symptom score in our study (i.e. the interaction effect remained after controlling for depressive and anxiety disorder symptoms). Hence, it might be that a more specific measure of irritability/sensitivity to threat [52] would have revealed an association. For the time being, this issue remains open and needs to be analyzed in future studies.

In our analyses, we considered indicators of ACE which have often been found to be associated with ADHD. The variables explained significant variance in HCC. In the ADHD group, children of mothers with a lower education level and higher depressive symptoms showed lower HCC. This corresponds to the findings of studies by White et al. [21, 53] on children/adolescents with externalizing disorders: low HCC was found in children with externalizing disorders who experienced (emotional) maltreatment. Maternal depressive symptoms may reflect family adversity and indicate less adequate parenting, thus potentially chronic ACE, which in turn leads to low HCC in the child [10]. Recent results of a longitudinal twin study confirmed a mediation process. Iob et al.[54] found basal cortisol to mediate 18% of the total association between dysfunctional parenting/emotional abuse (including maternal depression) and depressive symptoms in adolescence while controlling for genetic effects. Moreover, a twin study on adolescent HCC in particular pointed to non-shared environmental effects (69%) besides moderate heritability (39%) [55]. Hence, it is possible that environmental effects or gene-environment interaction effects explain our results. However, it is also possible that genetic effects, effects of fetal programming of the HPAA system [10, 32, 56], or severity of ADHD explain the association between maternal depressive symptoms and low HCC in the child. It is a limitation of our study that we cannot distinguish between these components. The associations should be analyzed in more detail in future studies as it is possible that specific environmental conditions are implicated in HPA axis dysfunction in ADHD subtypes.

Several strengths of the present study can be noted, including the analysis of medication-naïve children, the detailed assessment of confounders, the use of dimensional scores and diagnoses from well-validated clinical interviews, the use of an indicator of SR, and the use of HCC as a reliable and valid measure of the cumulated long-term activity of the HPAA. Limitations might be seen in the following issues: We found that low HCC relates to specific phenotypic characteristics of ADHD. Though compatible with current theorizing, the biological mechanisms linking HCC to inattentive symptom presentation in boys are largely unknown and should, therefore, be analyzed in the future. Moreover, indicators of ACE could have been analyzed in more detail. Considering maltreatment could be particularly elucidating (see e.g. [21, 53, 57]). Finally, in future studies, it may be illuminating to analyze further hormones (e.g. corticotropin-releasing factor and adrenocorticotropic hormone) and parameters (e.g. diurnal cortisol profile, cortisol reactivity and regulation) of the HPAA to determine which components of the system are affected.

To conclude, our findings underscore the presence of sex differences in the association of HCC with phenotypic features of ADHD. Indicators of ACE were related to low HCC in ADHD while comorbid conditions played an ancillary role. Hence, in the context of ADHD in childhood, HCC constitutes a meaningful marker of homogenous subgroups. It appears worthwhile to further analyze the role of HCC in ADHD in childhood. The marker might help to circumscribe specific developmental pathways and to provide tailored preventive measures and treatment approaches.

## Data Availability

The datasets generated during and/or analyzed during the current study are available from the corresponding author on reasonable request.
